# Centrosome associated genes pattern for risk sub-stratification in multiple myeloma

**DOI:** 10.1186/s12967-016-0906-9

**Published:** 2016-05-28

**Authors:** Fedor Kryukov, Pavel Nemec, Lenka Radova, Elena Kryukova, Samuel Okubote, Jiri Minarik, Zdena Stefanikova, Ludek Pour, Roman Hajek

**Affiliations:** Department of Haematooncology, Faculty of Medicine, University of Ostrava, Dvořákova 7, 702 00 Ostrava, Czech Republic; Department of Haematooncology, University Hospital Ostrava, 17.listopadu 1790, 708 52 Ostrava-Poruba, Czech Republic; Department of Biology, Faculty of Medicine, Masaryk University, Brno, Czech Republic; Department of Pathological Physiology, Faculty of Medicine, Masaryk University, Kamenice 5, 625 00 Brno, Czech Republic; The Central European Institute of Technology, Masaryk University, Kamenice 5, 625 00 Brno, Czech Republic; Department of Internal Medicine, University Hospital Olomouc, I.P. Pavlova 185/6, 779 00 Olomouc, Czech Republic; Department of Hematology and Blood Transfusion, University Hospital Bratislava, Antolská 11, 851 07 Bratislava, Slovak Republic; Department of Clinical Hematology, University Hospital Brno, Jihlavská 20, 625 00 Brno, Czech Republic

**Keywords:** Multiple myeloma, Gene expression profiling, Risk stratification

## Abstract

**Background:**

The genome of multiple myeloma (MM) cells is extremely unstable, characterized by a complex combination of structure and numerical abnormalities. It seems that there are several “myeloma subgroups” which differ in expression profile, clinical manifestations, prognoses and treatment response. In our previous work, the list of 35 candidate genes with a known role in carcinogenesis and associated with centrosome structure/function was used as a display of molecular heterogeneity with an impact in myeloma pathogenesis. The current study was devoted to establish a risk stratification model based on the aforementioned candidate genes.

**Methods:**

A total of 151 patients were included in this study. CD138+ cells were separated by magnetic-activated cell sorting (MACS). Gene expression profiling (GEP) and Interphase FISH with cytoplasmic immunoglobulin light chain staining (cIg FISH) were performed on plasma cells (PCs). All statistical analyses were performed using freeware R and its additional packages. Training and validation cohort includes 73 and 78 patients, respectively.

**Results:**

We have finally established a model that includes 12 selected genes (centrosome associated gene pattern, CAGP) which appears to be an independent prognostic factor for MM stratification. We have shown that the new CAGP model can sub-stratify prognosis in patients without TP53 loss as well as in IMWG high risk patients’ group.

**Conclusions:**

We assume that newly established risk stratification model complements the current prognostic panel used in multiple myeloma and refines the classification of patients in relation to the disease risks. This approach can be used independently as well as in combination with other factors.

**Electronic supplementary material:**

The online version of this article (doi:10.1186/s12967-016-0906-9) contains supplementary material, which is available to authorized users.

## Background

Multiple myeloma (MM) is a lymphoproliferative disease characterized by the clonal expansion of neoplastic plasma cells within the bone marrow. The genome of the malignant plasma cells is extremely unstable, characterized by a complex combination of structure and numerical abnormalities. These abnormalities serve as background for large variability in clinical course and outcome of MM patients [1]. A multistep process of malignant transformation can explain the presence of these genetic and clinical heterogeneities, which is admittedly associated with cell cycle deregulation.

Although several staging systems based on clinical and laboratory tests have been developed for MM [2, 3], standard prognostic factors, such as β2-microglobulin, albumin and C-reactive protein, account for only 15–20 per cent of outcome heterogeneity [4]. Previously, conventional cytogenetics was recognized as a relevant prognostic tool in multiple myeloma. Nevertheless, in spite of advances in molecular cytogenetics, many others undefined abnormalities forming genetic complexity in MM may still exist [5].

In our previous studies, we have used gene expression profiling to analyze a set of genes involved in formation of centrosome abnormalities in MM. Taking into consideration that centrosome amplification is common in all stages of plasma cell neoplasia [6] and is therefore an early event in MM and can serve as a source of genomic instability [7, 8]. Centrosome associated molecular signature is related to overall survival as well as to clinical parameters and ISS staging in MM [9]. We have identified a gene pattern, which was used as a display of molecular heterogeneity with an impact on myeloma pathogenesis [10].

We believe that the analysis of molecular signature will supplement existing prognostic models based on screening of chromosomal aberrations in plasma cells. Thus, the objective of our study was to create and validate risk stratification model based on previously described centrosome associated genes pattern in MM.

## Design and methods

### Patients and sample preparation

A total of 151 patients with MM enrolled in University Hospital Brno, Czech Republic, University Hospital Olomouc, Czech Republic and University Hospital Bratislava, Slovakia, were included in this study. The study was approved by the Ethical Committee of the Faculty of Medicine, Masaryk University (chairman: Josef Kure, PhD; ref number: 14/2009 and 29/2011), and the study was conducted according to the Helsinki declaration. All patients provided written informed consent. Training and validation cohort includes 73 and 78 patients, respectively. Patients’ baseline characteristics are summarized in Table 1.

**Table 1:** Patients’ baseline characteristic

	Training cohort^a^	Validation cohort^a^
No. of patients	73	78
Follow-up median (min–max) [month]	23.6 (0.3–97.0)	18.6 (0.1–250.0)
Gender: males–females	49.3–50.7 %	55.1–44.9 %
Age median (range) [years]	69 (38–84)	66 (40–90)
ISS stage: I–II–III	28.8 %–27.3 %–43.9 %	24.7 %–35.1 %– 40.3 %
Durie-Salmon stage: I–II–III	4.3 %–14.3 %–81.4 %	3.8 %–29.5 %–66.7 %
Durie-Salmon substage: A–B	81.4 %–18.6 %	75.6 %–24.4 %
Ig isotype: IgG–IgA–FLC-Non-secr.	60.3 %–23.5 %–16.2 %	57.7 %–28.2 %–1.3 %–12.8 %
Light chains: kapp–lambda	58.0 %–42.0 %	53.8 %–46.2 %
Plasma cell infiltration in bone marrow	34.4 % (0.8 %–93.6 %)	36.0 % (2.2 %–81.2 %)
No. of previous treatment lines		
None (first line treatment)	57.7 % (41/71)	64.1 % (50/78)
One	19.7 % (14/71)	15.4 % (12/78)
Two	8.5 % (6/71)	10.3 % (8/78)
More (>2)	14.1 % (10/71)	10.3 % (8/78)
Treatment regimen		
Bortezomib-based	47.8 % (32/67)	64.9 % (50/77)
Thalidomide-based	14.9 % (10/67)	10.4 % (8/77)
Lenalidomide-based	25.4 % (17/67)	18.2 % (14/77)
Others	11.9 % (8/67)	6.5 % (5/77)
Treatment response		
CR-VGPR-PR-MR-SD-PG	12.3 %–29.8 %–22.8 %–5.3 %–5.3 %–26.3 %	10.8 %–20.0 %–27.7 %–7.7 %–4.6 %–29.2 %
Biochemical parameters		
Hemoglobin (g/l)	105.5 (67.0–151.0)	95.5 (65.9–146.0)
Thrombocytes (×10^9^)	192.0 (33.0–416.0)	188.5 (55.0–485.0)
Calcium (mmol/l)	2.29 (1.74–23.37)	2.32 (1.75–2.78)
Albumin (g/l)	38.2 (21.1–54.1)	35.7 (17.4–52.2)
Creatinine (umol/l)	98.5 (53.0–783.0)	94.5 (30.0–849.0)
β_2_-microglobulin (mg/l)	4.70 (1.79–42.60)	4.61 (1.62–50.0)
Lactate dehydrogenase (ukat/l)	3.72 (1.53–22.92)	3.36 (1.14–7.77)
C-reactive protein (mg/l)	3.6 (0.0–174.3)	4.0 (0.0–149.3)
Monoclonal Ig (g/l)	29.8 (0.0–92.6)	30.2 (0.0–85.6)
Chromosomal abnormality		
Deletion 13q14	49.2 % (30/61)	60.0 % (39/65)
Deletion 17p13	8.3 % (5/60)	13.8 % (9/65)
Translocation t(4;14)	46.9 % (15/32)	44.4 % (16/36)
Amplification 1q21	56.9 % (37/65)	54.5 % (36/66)
Hyperdiploidy (H-MM)	45.5 % (30/66)	47.0 (17/46)

*CR* complete response; *VGPR* very good partial response; *PR* partial response; *MR* minimal response; *SD* stable disease; *PG* progression

^a^Both cohorts have no significant difference in basic clinical parameters

The bone marrow of patients was obtained during routine diagnostic procedure. Plasma cells in mononuclear cell fraction were enriched by anti-CD138+ immunomagnetic beads and sorted using AutoMACS (Miltenyi Biotec). Purity of CD138 + fraction was measured by flowcytometry and/or cytospin and samples with >80 % plasma cells were provided for total RNA isolation.

### Gene expression profiling (GEP)

Total RNA was isolated using QIAGEN RNeasy Mini Kit. RNA isolation. purification, and microarray hybridization has been reported previously [11]. Total RNA with purity ratio 260/280 >1.7 and integrity (RIN) >7.5 (as measured by Agilent 2100 Bioanalyzer) was transcribed into cDNA (Ambion WT Expression Kit), labeled and hybridized to the Affymetrix GeneChip^®^ Human Gene ST 1.0 array and further processed through R/Bioconductor framework by oligo package. RMA normalized data at gene level were statistically analyzed. Generated CEL files of patients included in this study have been deposited in the ArrayExpress Archive database under accession number E-MTAB-1038 for training set and E-MTAB-4032 for validation set. Both are available online (http://www.ebi.ac.uk/arrayexpress/).

### Fluorescence in situ hybridization (FISH)

FISH was performed as a part of routine diagnostic procedure as previously described [12]. The following aberrations were studied: 1q21 gain, 13q14 deletion, 17p13 deletion and translocation t(4;14). Hyperdiploidy status was determined with commercial probes mapping to chromosome 5 (LSI D5S23/D5S721), 9 (CEP9) and 15 (CEP15) (Abbott Molecular, Des Plaines, IL, USA).

### Statistical analysis

All statistical analyses were performed using freeware R and its additional packages: oligo, affy, survival, nnet and pROC [13–15]. Training and validation cohort includes 73 and 78 patients, respectively. Both cohorts have no significant differences in basic clinical parameters and all continuous variables were tested by nonparametric Mann–Whitney test. For categorical variables, the Fisher exact test was used. Overall survival (OS) was calculated from the date of diagnosis to death; progression free survival (PFS) was defined by the date of diagnosis and the date of disease progression or any death; time to progression (TTP) was defined by the date of diagnosis and the date of disease progression or disease-related death. Survival rates were estimated using the Kaplan–Meier method. Differences in survival of patients’ subgroups were compared using the log-rank test. p values below 0.05 were considered statistically significant.

## Results

In our previous study [9], a set of 111 genes with a known role in oncogenesis associated with centrosomal structure/function abnormalities corresponding to their proteins were selected for hierarchical clustering of gene expression (RMA-normalized, log 2 transformed expression level) profiles on CD138+ plasma cells from 73 patients with multiple myeloma. Furthermore, clustering analysis revealed a pattern of 35 genes. These genes and patients were later re-clustered to reveal three subgroups of patients according to different expression patterns of the chosen genes: “high expressed,” “medium expressed” and “low expressed,” respectively.

The list of 35 initially identified candidate genes were utilized for the generation of 3-subgroup predictor. To discover the gene signature that is able to predict subgroup membership of each sample, the bidirectional stepwise selection procedure with multinomial logistic regression model was performed. The best model was selected due to the Akaike information criteria. To apply such procedure, 12 candidate genes (*BUB1, BUB1B|PAK6, RAD51, PLK1, BRCA1, CENPA, BARD1, AURKA, MAD2L1, CENPH, XRCC2* and *CDC25C|FAM53C*) were identified and coefficients of multinomial regression model were estimated. The suggested regression model allows to predict the grouping of samples from both training and validation cohort into one of the subgroups (“high expressed,” “medium expressed” or “low expressed”). The pseudocode of predictive analyses and expression level of candidate genes are available in Additional files 1 and 2.

**Fig. 1:**
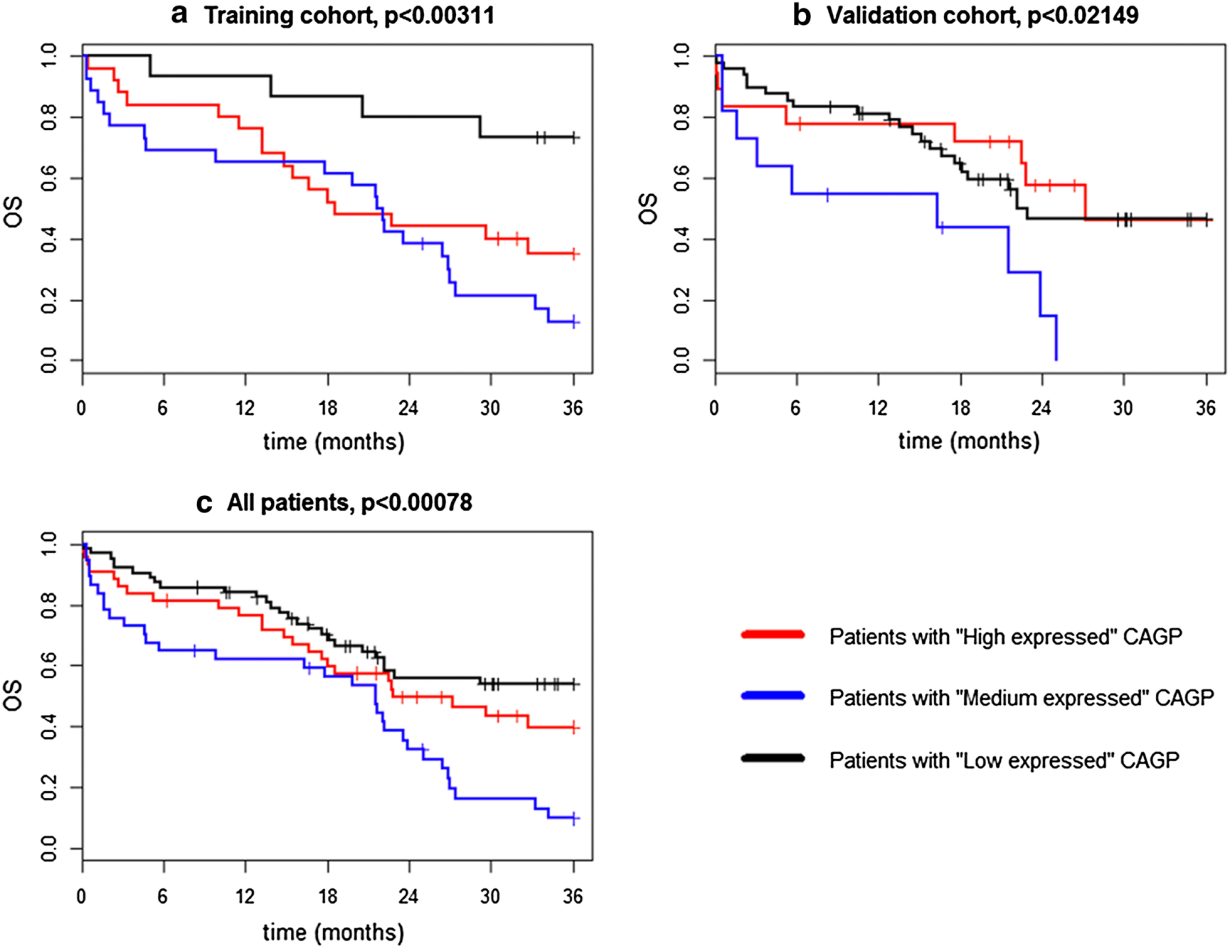
Overall survival of three CAGP expression groups of MM patients. **a** Kaplan–Meier curves for OS of training patients’ cohort (n = 73) stratified by centrosome associated gene pattern (CAGP). **b** Kaplan–Meier curves for OS of validation patients’ cohort (n = 78) stratified by centrosome associated gene pattern (CAGP). **c** Kaplan–Meier curves for OS of jointed training and validation patients’ cohort (n = 151) stratified by centrosome associated gene pattern (CAGP)

Based on this centrosome associated genes pattern (CAGP) model, patients were stratified into three groups—“High expressed”, “Medium expressed” and “Low expressed”. The overall survival of patients in “High expressed” and “Medium expressed” subgroups was significantly worse than in patients in “Low expressed” subgroup for both training and validation cohorts (Fig. 1) and was in concordance with previously published results [9]. Analysis of progression free survival (PFS) and time to progression (TTP) in the three CAGP expression groups did not reveal any significant differences (data not shown).

**Fig. 2:**
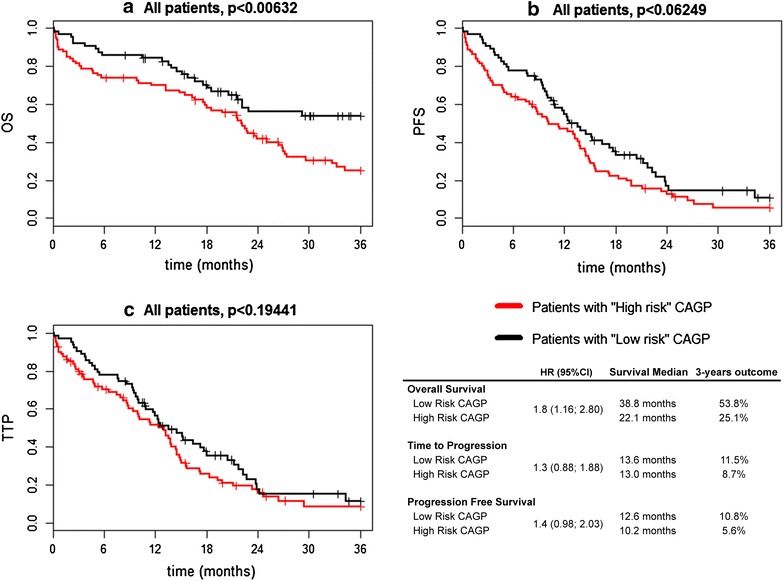
Overall survival, progression free survival and time to progression in CAGP risk groups of MM patients. **a** Kaplan–Meier curves for OS of combined training and validation patients’ cohort (n = 151) stratified by centrosome associated gene pattern (CAGP). **b** Kaplan–Meier curves for PFS of combined training and validation patients’ cohort (n = 151) stratified by centrosome associated gene pattern (CAGP). **c** Kaplan–Meier curves for TTP of combined training and validation patients’ cohort (n = 151) stratified by centrosome associated gene pattern (CAGP)

Additionally, we combined patients with worse overall survival and clinical characteristics in High- and Medium- expressed subgroups as “HR CAGP” (high risk centrosome associated genes pattern). Patients with Low expressed were defined as “LR CAGP” (low risk centrosome associated genes pattern), respectively. Significantly worse prognosis was found for HR CAGP group (HR = 1.8; 3-year OS = 25.1 %) compared to LR CAGP group (3-year OS = 53.8 %, p < 0.01). Analysis of PFS and TTP in two CAGR risk groups did not reveal any significant differences (Fig. 2).

To characterize the prognostic significance of CAGP model, multivariate Cox proportional hazards survival model was used. Besides CAGP, the following parameters were used for multivariate Cox proportional hazards survival model: ISS stage, β2-microglobulin, del 17p13, t(4;14), amp 1q21. The variables in the multivariate model were the only variables, which remained statistically significant when potential predictors were combined together including CAGP, which was forced into the model. Among all subsequently tested combinations of predictors, the best results in risk of death assessment were obtained for CAGP combined with del 17p13 (p < 0.001). It is worth to mention that both prognostic factors were independent. “HR” CAGP subgroup as well as TP53 deletion had significantly higher risk of death assessment (HR = 1.8 and HR = 2.5, respectively; p < 0.005). Survival characteristics for different risk sub-groups are presented in Table 2. Apparent controversies in sub-stratified TP53 deletion groups probably associated with small cohort size (7 patients in each TP53+/low risk CAGP and TP53+/high risk CAGP sub-groups).

**Table 2:** Survival characteristics for different TP53 risk groups sub-stratified with CAGP model

Stratification group	N	HR (95 % CI)	Survival median (months)	3-years outcome (%)	p value (log-rank)
Overall survival					
Low risk CAGP	66		38.8	53.8	<0.00799
High risk CAGP	84	1.8 (1.16; 2.80)	22.1	25.1	
TP53−	110		25.1	35.6	<0.00281
TP53+	14	2.5 (1.35; 4.73)	12.8	12.2	
TP53−/low risk CAGP	47		38.8	61.1	<0.00099
TP53−/high Risk CAGP	63	2.5 (1.41; 4.30)	22.2	20.1	
TP53+/low Risk CAGP	7		12.8	0	<0.3621
TP53+/high risk CAGP	7	0.5 (0.14; 2.05)	16.7	21.4	
Time to progression					
Low risk CAGP	66		13.6	11.5	<0.1939
High risk CAGP	84	1.3 (0.88; 1.88)	13.0	8.7	
TP53−	110		13.9	11.6	<0.01378
TP53+	14	2.1 (1.15; 3.79)	8.7	0	
TP53−/low risk CAGP	47		16.4	15.3	<0.08914
TP53−/high risk CAGP	63	1.5 (0.94; 2.34)	13.2	8.0	
TP53+/low risk CAGP	7		7.6	0	<0.676
TP53+/high risk CAGP	7	0.8 (0.23; 2.61)	9.9	0	
Progression free survival					
Low risk CAGP	66		12.6	10.8	<0.06232
High risk CAGP	84	1.4 (0.98; 2.03)	10.2	5.6	
TP53−	110		13.2	8.8	<0.04351
TP53+	14	1.8 (1.01; 3.29)	8.7	0	
TP53−/low risk CAGP	47		15.2	14.1	<0.02102
TP53−/high risk CAGP	63	1.7 (1.07; 2.55)	11.3	4.6	
TP53+/low risk CAGP	7		7.6	0	<0.676
TP53+/high risk CAGP	7	0.8 (0.23; 2.61)	9.9	0	

Low risk CAGP” group includes patients with “Low expressed” centrosome associated gene pattern. “High risk CAGP” group includes patients with united “High and medium expressed” centrosome associated gene pattern. “TP53+” group includes patients with deletion 17p13; “TP53−” group includes patients without deletion 17p13 (positivity cut-off >20 %)

Further analysis includes comprising of CAGP model and International Myeloma Working Group (IMWG) risk stratification model [16, 17]. Briefly, IMWG consensus recommendations includes the following makers: serum β2-microglobulin, serum albumin, t(4;14), 17p13 and 1q21 by FISH. Using this combination, high-risk patients will survive less than 2 years despite novel agents, and low-risk patients can survive for more than 10 years [16]. In total, 70 patients had sufficient clinical data to be included to the analysis. To the IMWG High and Standard Risk groups belong 28 and 41 patients respectively. The only one patient belongs to IMWG Low Risk group was excluded from the further analysis.

Fisher exact test did not show significance of the association (contingency) between the two kinds of risk classification (p = 0.55). Thus, CAGP model can be used to sub-stratify IMWG risk groups. Survival characteristics for Standard and High Risk IMWG groups sub-stratified with CAGP model are presented in Table 3.

**Table 3:** Survival characteristics for standard and high risk IMWG groups sub-stratified with CAGP model

Stratification group	N	HR (95 % CI)	Survival median (months)	3-years outcome (%)	*p* value (log-rank)
Overall survival					
Low risk CAGP	66		38.8	53.8	<0.00799
High risk CAGP	84	1.8 (1.16; 2.80)	22.1	25.1	
IMWG standard risk	41		22.8	32.1	<0.1371
IMWG high risk	28	1.6 (0.86; 2.91)	15.8	23.5	
IMWG standard risk/low risk CAGP	18		29.2	42.9	<0.4532
IMWG standard risk/high risk CAGP	23	1.4 (0.59; 3.25)	22.8	27.4	
IMWG high risk/low risk CAGP	14		16.6	49.9	<0.02836
IMWG high risk/high risk CAGP	14	2.8 (1.07; 7.45)	11.5	8.2	
Time to progression					
Low risk CAGP	66		13.6	11.5	<0.1939
High risk CAGP	84	1.3 (0.88; 1.88)	13.0	8.7	
IMWG standard risk	41		13.2	6.9	<0.7824
IMWG high risk	28	1.1 (0.62; 1.89)	12.0	0	
IMWG Standard risk/low risk CAGP	18		12.3	0	<0.9978
IMWG standard risk/high risk CAGP	23	1 (0.48; 2.07)	14.5	9.6	
IMWG high risk/low risk CAGP	14		17.7	0	<0.05469
IMWG high risk/high risk CAGP	14	2.3 (0.96; 5.55)	8.0	0	
Progression free survival					
Low risk CAGP	66		12.6	10.8	<0.06232
High risk CAGP	84	1.4 (0.98; 2.03)	10.2	5.6	
IMWG standard risk	41		13.2	6.3	<0.8121
IMWG high risk	28	1.1 (0.62; 1.84)	11.4	0	
IMWG standard risk/low risk CAGP	18		12.3	0	<0.9395
IMWG standard Risk/high risk CAGP	23	1 (0.48; 1.97)	13.9	9.1	
IMWG high risk/low risk CAGP	14		17.7	0	<0.0325
IMWG high risk/high risk CAGP	14	2.5 (1.05; 5.89)	7.7	0	

“Low risk CAGP” group includes patients with “Low expressed” centrosome associated gene pattern. “High risk CAGP” group includes patients with united “High and medium expressed” centrosome associated gene pattern. “IMWG standard risk” group includes patients with ISS III and no adverse FISH or ISS I and t(4;14)/17p13 del; “IMWG high risk” group includes patients with ISS II/III and t(4;14)/17p13 del

Data shows that proposed CAGP model can be used to sub-stratify IMWG High risk group. This statement is relevant for overall survival, progression-free survival and time to progression.

## Discussion

The role of genes is a potential for the determination of molecular signature as a genetic based prognostic or predictive marker, but it is still currently unclear. High- and low-risk groups defined with cytogenetic prognostic models that is based on the most important chromosomal abnormalities such as deletion 17p13 (TP53 gene), translocation t (4;14) and gain 1q21, are still heterogeneous [18, 19]. Heterogeneity determined by molecular variability also determines the diversity of disease at clinical level. It seems that there are several “myeloma subgroups” which differ in expression profile and in clinical manifestations, i.e., prognosis and response for treatment [20]. Until now, approximately 10 % of low-risk patients relapse in 2 years whereas a reverse tendency can be observed in high-risk group as 5–10 % do not reach early relapse (our unpublished data). In spite of substantial progress in therapeutics, the outcome for patients requiring therapy is still highly variable.

In our previous work, we identified the list of 35 candidate genes that play a known role in carcinogenesis and associated with centrosome structure/function, which was used to display molecular heterogeneity with an impact on myeloma pathogenesis [9]. The current study was devoted to create and validate risk stratification model based on these centrosome associated candidate genes. Finally, the created model including 12 selected genes (centrosome associated gene pattern) appears to be an independent prognostic factor for MM stratification.

Nowadays, however, it is needed to use an integrated genomics approach to develop a comprehensive model for risk stratification [21], as GEP-based signature alone appears to have limited power for prognosis in MM [22]. We believe that stratification models reflecting RNA level (gene expression profiling) can supply stratification models reflecting DNA level (interphase fluorescence in situ hybridization).

We have shown that the combination of two independent risk factors such as expression of centrosome associated related genes pattern (CAGP) with TP53 loss depicts the best results in death assessment risk stratification. We suppose that the stated combination of risk factors has become pathogenetically relevant. It may be logically explained by affecting the centrosome associated mitotic damage, which appears catastrophic in the absence of p53-dependent checkpoint response [23, 24].

Summarizing our previously published studies [25], we suggest that centrosome dysfunction accomplished with safe apoptotic system will reset cell cycle and make such clone more sensitive to pro-apoptotic signals. In contrast, in case of an affected apoptotic response, centrosome dysfunction will cause severe genomic instability, which evade apoptosis despite being induced, and may eventually develop a clone with even more aggressive phenotype. Probably, systemic study of apoptotic response in concordances with integrative “omics” study of centrosome machinery will elucidate biological background of revealed controversies in sub-stratified TP53 loss groups.

In conclusion, we have created a new GEP-based model for classifying every patient into one of two prognostic subgroups (high- and low risk CAGP). It seems that CAGP model is able to sub-stratify TP53 negative subgroup: patients with high expressed CAGP attribute to the group with higher risk of death assessment, while patients with low expressed CAGP attribute to the group with lower risk of death assessment within aforementioned subgroup.

Basic laboratory tests including serum albumin and β2-microglobulin for ISS staging, and FISH for t(4;14), del 17p13 and 1q21 gain are markers risk stratification themselves [17, 26]. International Myeloma Working Group consensus on risk stratification in multiple myeloma recommends combination of ISS and FISH for risk stratification. This risk model includes serum β2-microglobulin, serum albumin, t (4;14), 17p13 and 1q21 by FISH [16]. IMWG High Risk group has surviving less than 2 years despite novel agents and includes patients with ISS II/III and t (4;14)/17p13 del. IMWG Standard Risk group includes patients with ISS III and no adverse FISH or ISS I and t (4;14)/17p13 del. IMWG Low Risk group can survive for more than 10 years and includes ISS I/II without adverse FISH [16, 17]. In our study, cohort of patients from IMWG high risk group has overall survival median 15.8 month with 3-year survival 23.5 % and corresponding with previously published data [16]. We found no significant association between the IMWG and CAGP risk classifications. Moreover, CAGP model can be used to sub-stratify IMWG High Risk group: patients with high expressed CAGP attribute to the group with higher risk of death assessment, shorter overall survival, progression-free survival and time to progression.

## Conclusions

We assume that the newly established prognostic stratification model complements the current prognostic panel used in multiple myeloma and refines the classification of patients in relation to the risk of disease. This approach can be used independently as well as in combination with other factors. Thus, the new model can sub-stratify prognosis in patients without TP53 loss as well as in IMWG high risk patients’ group. These findings need to be confirmed on a larger cohort with longer follow-up.

## Additional files

10.1186/s12967-016-0906-9 Centrosome associated gene pattern (CAGP) expression subgroups.
10.1186/s12967-016-0906-9 Pseudocode of predictive analyses.

## Abbreviations

MM: multiple myeloma
GEP: gene expression profiling
FISH: fluorescent in situ hybridization
CAGP: centrosome associated gene pattern
IMWG: International Myeloma Working Group
OS: overall survival
PFS: progression free survival
TTP: time to progression
ISS: international staging system
