# Supplementary figures and images for: Centrosome associated genes pattern for risk sub-stratification in multiple myeloma
Source: J Transl Med. 2016 May 28;14:150. doi: 10.1186/s12967-016-0906-9 (PMC4884414; doi:10.1186/s12967-016-0906-9)

**BARD1**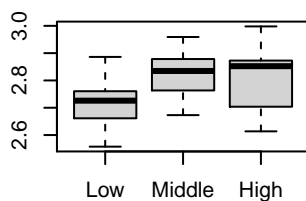**CDC25C.FAM53C**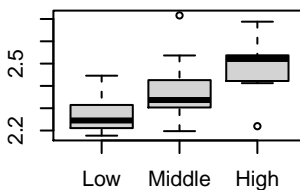**MAD2L1**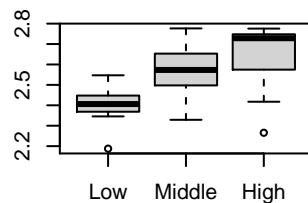**CENPA**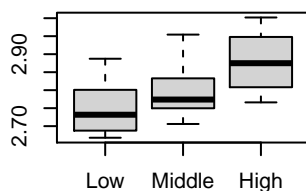**RAD51**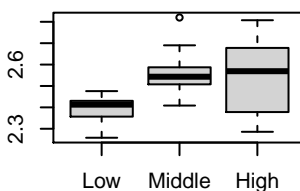**BUB1B.PAK6**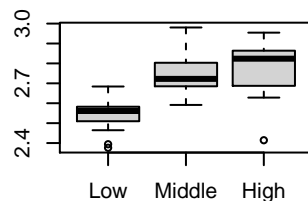**CENPH**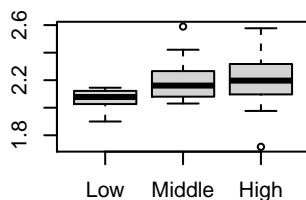**XRCC2**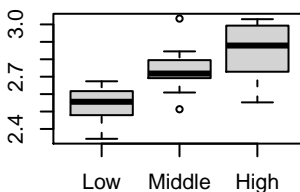**PLK1**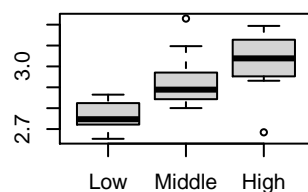**BRCA1**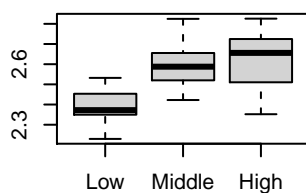**BUB1**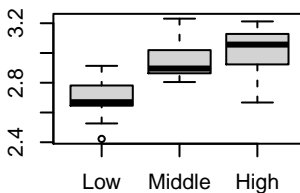**AURKA**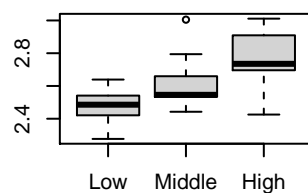

Supplement: Supplementary file 1 — 10.1186/s12967-016-0906-9 Centrosome associated gene pattern (CAGP) expression subgroups. [file 12967_2016_906_MOESM1_ESM.pdf]
