# Supplementary material for: Centrosome associated genes pattern for risk sub-stratification in multiple myeloma
Source: J Transl Med. 2016 May 28;14:150. doi: 10.1186/s12967-016-0906-9 (PMC4884414; doi:10.1186/s12967-016-0906-9)
Supplement: Supplementary file 2 — 10.1186/s12967-016-0906-9 Pseudocode of predictive analyses. [file 12967_2016_906_MOESM2_ESM.docx]

**Pseudocode of predictive analyses**

1. TR_eSet = load expression set E-MTAB-1038 including gene names and subgroup information (“low-expressed”, “middle-expressed”, “high-exressed”)
2. VALID_eSet = load validation expression set including gene names
3. TRAINING = subset of TR_eSet with 35 genes from Table II in ([Kryukov, Nemec et al. 2013](#_ENREF_1))
4. MODEL = results of bidirectional stepwise selection for multinomial model applied on dataset TRAINING (model with minimal Akaike information criteria)
5. GENES_FINAL = genes in MODEL (12 genes listed in main text)
6. PRED_GROUP = individual predictions of MODEL for samples in VALID_eSet and predictors GENES_FINAL
7. multiclass ROC analyses for both TRAINING and VALID_eSet due to the MODEL

Generated CEL files of patients included in this study have been deposited in the ArrayExpress Archive database under accession number E-MTAB-1038 for training set and E-MTAB-4032 for validation set. Both are available online [(http://www.ebi.ac.uk/arrayexpress/).](http://www.ebi.ac.uk/arrayexpress/)

**References**

Kryukov, F., P. Nemec, E. Dementyeva, L. Kubiczkova, I. Ihnatova, E. Budinska, J. Jarkovsky, S. Sevcikova, P. Kuglik and R. Hajek (2013). "Molecular heterogeneity and centrosome-associated genes in multiple myeloma." Leuk Lymphoma **54**(9): 1982-1988.
